# Comparing effects: a reanalysis of two studies on season of birth bias in anorexia nervosa

**DOI:** 10.1186/s40337-016-0131-1

**Published:** 2017-01-10

**Authors:** Eirin Winje, Anne-Kari Torgalsbøen, Cathrine Brunborg, Kristin Stedal

**Affiliations:** 1 Division of Mental Health and Addiction, Regional Department for Eating Disorders, Oslo universitetssykehus HF, Postboks 4956, Nydalen, 0424 Oslo, Norway; 2 Department of Psychology, University of Oslo, P.O. Box 1039, 0317 Oslo, Norway; 3 Oslo Centre for Biostatistics and Epidemiology, Research Support Services, Oslo University Hospital, Ullevål, Oslo, Norway

**Keywords:** Season of birth, Methodology, Anorexia nervosa, Eating disorders, Effect sizes

## Abstract

**Background:**

Outcomes from studies on season of birth bias in eating disorders have been inconsistent. This inconsistency has been explained by differences in methodologies resulting in different types of effect sizes. The aim of the current study was to facilitate comparison by using the same methodology on samples from two studies with differing conclusions.

**Methods:**

The statistical analyses used in each study were applied to the samples from the other study and the resulting effect sizes, Cramêr’s V and odds ratio (OR), were compared and discussed.

**Results:**

For both studies, the Cramêr’s Vs ranged between 0.03 and 0.08 and the OR ranged between 0.85 and 1.31. According to common conventions, Cramêr’s Vs below 0.10 and ORs below 1.44 are considered small.

**Conclusion:**

As a marker of one or more potential risk factors, the observed effects are considered to be small. When reanalysed allowing for direct comparisons, studies with contrasting conclusions converge towards an absence of support for a season of birth bias for patients with AN.

## Background

A season of birth bias means that more patients than expected from the normal population are born during certain months; indicating this could be a marker of yet unknown causal factors for that disorder [1, 2]. Several studies have investigated season of birth bias in eating disorders [1–4]. However, the conclusions from the two largest studies in the field have been contrasting. Disanto and colleages [1] concluded that there was a significant season of birth bias for patients with anorexia nervosa (AN). Their sample consisted of patients with AN collected from four previously published studies [3–6], and was compared to the national distribution of births retrieved from the UK Office for National Statistics. On the other hand, Winje and colleagues [2] concluded that their findings did not support a season of birth bias hypothesis. Their sample consisted of females with AN who were recruited from 16 centres in nine different countries, resulting in five samples which were compared to the distribution of births in the general population in the same areas, retrieved from the corresponding statistical bureaus.

It has been proposed that the inconsistent findings could be due to either a lack of sufficient statistical power to detect small differences, or because of differences in statistical methods [1]. The former increases the risk for Type II errors, and the latter complicates comparisons between studies, as different methods produces different types of effect sizes [7]. In addition, previous studies have not defined a priori which effect size which would be theoretical or clinical interesting. Further, the observed effects have not been discussed in terms of their theoretical or clinical significance. This discussion is vital, since interpreting the magnitude of an effect allows us to understand the theoretical and clinical impact of a statistically significant finding [7].

## Methods

The aim of the current study was to facilitate direct comparison of effect sizes of the same type to investigate whether studies with contrasting conclusions can have similar findings. The studies by Disanto et al. [1] and Winje et al. [2] were chosen as i) their conclusions differ, ii) they both have large samples and included information on power calculations, iii) Disanto et al. [1] performed a meta-analysis (analysing the pooled sample), and iv) Winje et al. [2] included samples from several continents on both the Northern and the Southern hemisphere, as well as a pooled analysis. A secondary aim or this paper is to discuss the results according to common conventions for interpreting effect sizes (Cohen’s categories [8]) and their practical implications.

To enable comparison of the studies by Disanto and colleagues [1] and Winje et al. [2], the statistical analyses used in each study were applied to the samples from the other study. Disanto et al. [1] performed a Walter and Elwood’s test [9] and chi-square analyses contrasting i) the first vs. the second half of the year (1df), ii) March-June vs. the rest of the year (1df), iii) September-October vs. the rest of the year (1df), and iv) March-June vs. September- October (1df). The effects reported were odds ratios (OR). OR can be used in the context of binary categorical outcomes. It describes the odds of being in one group relative to the odds of being in a different group. It ranges from zero to infinitive, with an OR of 1 meaning no difference between the groups, OR >1 indicating an increase in odds relative to the reference group, and OR < 1 indicating a decrease.

Winje et al. [2] performed a two-tailed chi-square test for contingency tables with known population parameter [10] to test for monthly deviations (11 df). The effect sizes reported were Cramêr’s V. This is a measure of the inter-correlation between variables, when there are more than two categories. It can be interpreted like Pearson’s r and R^2^.

The chi-square tests are based on a test statistic that measures the divergence of the observed data from the values that would be *expected* under the null hypothesis. As Chi-square analyses are measures of association, causation cannot be inferred. The tests are of limited use if 20% if the expected values in any cell are less than 5, or the individual observations are not independent [10]. However, none of the expected values in this reanalysis had frequencies less than 5, and all the observations were independent.

For further details about the samples and the analyses, including power analyses, the reader is referred to the original papers [1, 2].

To allow for comparison of the effect sizes between the two papers, ORs were calculated in Vassarstat (http://vassarstats.net/odds2x2.html) for the samples from the study by Winje et al. [2]. Cramêr’s Vs were calculated in PASW 18 statistical software for the sample in the study by Disanto et al. [1]. The distribution for both the patients and the general populations in the study by Winje and colleagues [2] were retrieved from the original paper. The distribution for the samples that comprised the patients in the study by Disanto et al. [1] were retrieved from their source papers and the control data from the UK office for National Statistics. The samples in this study are subjected to multiple testing of the same hypothesis which raises the probability of type I errors. Thus, the predetermined statistical significance level (alpha-level) was adjusted accordingly. The conventional alpha-level of .05 was divided with the number of tests each sample was subjected to. The adjusted alpha-levels for Disanto and colleagues’ [1] sample was .01. In the study by Winje and colleagues’ [2] the alpha-level was 0.003 for sample i & ii and 0.005 for samples iii, iv and v.

## Results

The reanalyses demonstrate that the Cramêr’s V for both studies ranges from 0.03 to 0.08. The OR for all samples ranges from 0.85 to 1.31. Contrary to the findings by Disanto et al. [1], the observed confidence intervals for the ORs for Winje and colleagues’ samples [2] include 1 and the *p*-values do not reach statistical significance.

Table 1 displays the results from the reanalyses, the original findings from the study by Disanto and colleagues [1], and the original findings from the study by Winje et al. [2].

**Table 1:** Cramêr’s Vs and odds ratios for the reanalysed studies

Sample origin	Sample N	Monthly deviations (11 df)	First vs. second half of the year (1 df)	March–June vs. the rest of the year (1 df)	September–October vs. the rest of the year (1 df)	March–June vs. September–October (1 df)
Disanto et al. [1]						
UK	1239	Cramêr’s V (C) = 0.06^a^ *X* ^2^ = 10.6 *P* = 0.49	Odds ratio (OR) = 1.13 95% Confidence interval (CI) = 1.01–1.26 *P* = 0.025	OR = 1.15 95% CI = 1.03–1.29 *P* = 0.012	OR = 0.80 95% CI = 0.68–0.94 *P* = 0.007	OR = 1.31 95% CI = 1.10–1.56 *P* = 0.001
Winje et al. [2]						
i) Iceland, Norway, Sweden	815	C = 0.05 *X* ^2^ = 4.60 *P* = 0.94	OR = 0.96^a^ 95% CI = 0.79–1.16 *P* = 0.68	OR = 1.04^a^ 95% CI = 0.86–1.27 *P* = 0.67	OR = 1.00^a^ 95% CI = 0.77–1.30 *P* = 1	OR = 0.97^a^ 95% CI = 0.73–1.30 *P* = 0.84
ii) UK	706	C = 0.05 *X* ^2^ = 2.85 *P* = 0.99	OR = 1.10^a^ 95% CI = 0.90–1.36 *P* = 0.34	OR = 0.94^a^ 95% CI = 0.76–1.17 *P* = 0.60	OR = 0.99^a^ 95% CI = 0.75 *P* = 0.92	OR = 1.03^a^ 95% CI = 0.76–1.41 *P* = 0.84
iii) Oregon, USA	394	C = 0.07 *X* ^2^ = 3.93 *P* = 0.97	OR = 1.00^a^ 95% CI = 0.76–1.33 *P* = 1	OR = 1.06^a^ 95% CI = 0.79–1.43 *P* = 0.69	OR = 0.01^a^ 95% CI = 0.63–1.32 *P* = 0.67	OR = 0.89^a^ 95% CI = 0.59–1.34 *P* = 0.57
iv) Australia	382	C = 0.08 *X* ^2^ = 4.90 *P* = 0.94	OR = 1.11^a^ 95% CI = 0.83–1.47 *P* = 0.49	OR = 0.85^a^ 95% CI = 0.63–1.15 *P* = 0.29	OR = 1.17^a^ 95% CI = 0.80–1.73 *P* = 0.42	OR = 1.27^a^ 95% CI = 0.83–1.95 *P* = 0.27
v) Brazil, Argentina	485	C = 0.06 *X* ^2^ = 3.65 *P* = 0.98	OR = 0.96^a^ 95% CI = 0.75–1.24 *P* = 0.75	OR = 1.00^a^ 95% CI = 0.76–1.31 *P* = 1	OR = 0.93^a^ 95% CI = 0.67–1.30 *P* = 0.68	OR = 0.94^a^ 95% CI = 0.65–1.37 *P* = 0.76
Europa (i & ii)	1521	C = 0.03 *X* ^2^ = 2.16 *P* = 0.99	OR = 1.02^a^ 95% CI = 0.89–1.18 *P* = 0.74	OR = 0.99^a^ 95% CI = 0.86–1.16 *P* = 1	OR = 1.00^a^ 95% CI = 0.82–1.20 *P* = 1	OR = 1.00^a^ 95% CI = 0.81–1.23 *P* = 1
Northern Hemisphere (i, ii & iii)	1915	C = 0.03 *X* ^2^ = 3.03 *P* = 0.99	OR = 1.02^a^ 95% CI = 0.90–1.16 *P* = 0.76	OR = 1.01^a^ 95% CI = 0.88–1.15 *P* = 0.88	OR = 0.98^a^ 95% CI = 0.82–1.16 *P* = 0.77	OR = 0.96^a^ 95% CI = 0.80–1.16 *P* = 0.68
Southern Hemisphere (iv & v)	879	C = 0.04 *X* ^2^ = 2.52 *P* = 0.99	OR = 1.02^a^ 95% CI = 0.84–1.23 *P* = 0.89	OR = 0.93^a^ 95% CI = 0.76–1.14 *P* = 0.48	OR = 1.03^a^ 95% CI = 0.80–1.32 *P* = 0.82	OR = 1.07^a^ 95% CI = 0.81–1.42 *P* = 0.62

*Note:* ^a^Results from the reanalysis, the rest of the results are retrieved from the two original studies [1, 2]

## Discussion

To facilitate comparison across studies on season of birth in AN, the aim of the current study was to reanalyse the two largest studies to date in the field. The findings suggest that although the conclusions from previous studies differ, the effect sizes do not.

According to common conventions for interpreting effect sizes [8, 11], Cramêr’s Vs below 0.10 and OR below 1.44 are considered small. All the observed Cramêr’s Vs and the ORs in the original papers and the reanalyses, are below these cut-offs. Although most of the ORs observed for the samples in the study by Winje et al. [2] fluctuate close to 1 (no effect), the ORs reported in the paper by Disanto and colleagues’ [1] are not clinically significantly larger as they are all below the 1.44 cut-off for small effects, indicating less than 1% explained variance.

Only two ORs from the original study by Disanto and colleagues’ [1] had p-values below the predetermined alpha level, meaning that the results were unlikely if there were no underlying differences between the samples. However, the impact of any statistical significant findings is dependent on the interpretation of the effect sizes [7]. In this case, all the ORs were approximately similar in size to those observed in the reanalysis of the samples in the study by Winje et al. [2]. The remaining analyses would obtain lower p-values by increasing their sample sizes, as the p-value is a confounded index by being dependent on both the effect and sample size [8].

The applied contribution of season of birth research is to inform hypotheses of possible risk factors for AN. When determining if the observed effects in the current study are large enough to do this, at least two points are relevant. Firstly, chi-square analyses collapse any monthly deviations across the normal population and patients with AN. This means that the observed effects could be located in one month or distributed across the different months included in each analysis. This would yield even smaller effect sizes. Secondly, eating disorders are variable in onset and episodic in nature and different sets of risk factors might therefore be linked to onset, remission and relapse [12]. A season of birth bias could be a marker for one or more such risk factors. If so, it would be those other factors associated with the potential bias that would contribute to the development of AN, not the month/season of birth in itself [12]. Further, the findings from the current study show that if a correct effect size (Cramer’s V) is used on the 12 month comparison, there is good concordance between the Disanto et al. [1] results and all the Winje et al. [2] results, indicating that there is no evidence supporting a strong annual pattern of births differentiating patients with AN from healthy controls. As always, this of course does not prove that there is no such pattern; it may simply be very weak. Therefore, the potential gain in explanatory value from season of birth research needs to be compared to research focusing on other proposed risk factors.

The current study is limited by the possibility of sampling issues from the source studies. Both Disanto and colleagues [1] and Winje et al. [2] sampled different populations – either from different papers [1] or from different centres [2]. This creates the possibility of sampling problems (Simpson’s Paradox) which can influence the validity of the two original studies, and therefore also of the current study. Further, information regarding the diagnostic procedures leading to each individual’s inclusion or exclusion in its source study is unknown. This study also carries the limitation of not having defined a priori the theoretical or clinical significant effect size. In addition, the use of the Walter and Elwood test causes some concerns. This test requires for the researcher to have knowledge of the number of births for each month, and out of that number, note how many go on to develop AN. In other words, the Walter and Elwood test compares the prevalence in the various months and would therefore require a prospective study commencing at birth. However, in the source study [1] it is employed on retrospective data, collected from records. As the aim of the current study was to compare findings by applying the statistical methods used in the source studies, the appropriate test for this kind of research – the 11x2 Chi Square test used by Winje and colleagues – is employed for analysing both samples and thus allows for comparison of the two types of effects.

## Conclusion

In conclusion, when reanalysed allowing for comparison of effect sizes, well-powered studies with apparently inconsistent findings and contrasting conclusions converge towards an absence of support for a season of birth bias for patients with AN, indicating that the annual effect is either very small and perhaps non-existent.
